# Risk factors for breakthrough SARS-CoV-2 infection in vaccinated healthcare workers

**DOI:** 10.1371/journal.pone.0258820

**Published:** 2021-10-15

**Authors:** Moza Alishaq, Hanaa Nafady-Hego, Andrew Jeremijenko, Jameela Ali Al Ajmi, Mohamed Elgendy, Suni Vinoy, Sameera Bihi Fareh, Justine Veronica Plaatjies, Mariam Nooh, Nadya Alanzi, Anvar H. Kaleeckal, Ali Nizar Latif, Peter Coyle, Hamed Elgendy, Abdul-Badi Abou-Samra, Adeel Ajwad Butt

**Affiliations:** 1 Hamad Medical Corporation, Doha, Qatar; 2 Microbiology and Immunology Department, Faculty of Medicine, Assiut University, Assiut, Egypt; 3 Faculty of Medicine, Universiti Sains of Malaysia, Kelantan, Malaysia; 4 Weill Cornell Medical College, New York, NY, United States of America and Doha, Qatar; 5 Anesthesia Department, Faculty of Medicine, Assiut University, Assiut, Egypt; Istanbul University Istanbul Faculty of Medicine: Istanbul Universitesi Istanbul Tip Fakultesi, TURKEY

## Abstract

**Background and objective:**

The risk factors for breakthrough infections among healthcare workers (HCW) after completion of a full course of vaccination are poorly understood. Our objective was to determine the risk factors for breakthrough SARS-CoV-2 infection among HCWs at a national healthcare system in Qatar.

**Methods:**

We identified all HCWs at Hamad Medical Corporation in Qatar between December 20, 2020 and May 18, 2021 with confirmed SARS-CoV-2 RT-PCR infection >14 days after the second vaccine dose. For each case thus identified, we identified one control with a negative test after December 20, 2020, matched on age, sex, nationality, job family and date of SARS-CoV-2 testing. We excluded those with a prior positive test and temporary workers. We used Cox regression analysis to determine factors associated with breakthrough infection.

**Results:**

Among 22,247 fully vaccinated HCW, we identified 164 HCW who had breakthrough infection and matched them to 164 controls to determine the factors associated with SARS-CoV-2 breakthrough infection. In the breakthrough infection group the nursing and midwifery job family constituted the largest group, spouse was identified as the most common positive contact followed by a patient. Exposure to a confirmed case, presence of symptoms and all other job families except Allied Health Professionals when compared with nursing and Midwifery staff independently predicted infection.

**Conclusion:**

Presence of symptoms and contact with a confirmed case are major risk factors for breakthrough SARS-CoV-2 infection after vaccination, and these groups should be prioritized for screening even after full vaccination.

## Introduction

We recently reported the rate and risk factors for breakthrough SARS-CoV-2 infection in a high risk population is recently reported from the US Department of Veterans Affairs’ healthcare system [1]. Little is known about the risk factors for breakthrough infections among healthcare workers (HCW) who have been fully vaccinated. In addition to the risk of infection in the community settings, HCWs are potentially at a higher risk of infection due to prolonged and/or repeated exposure to hospitalized infected patients. The rate of SARS-CoV-2 infection in HCWs is reported to vary between 3 and 17% and varies according to the history and degree of exposure and presence of symptoms [2]. SARS-CoV-2 vaccination campaign in Qatar started in mid-December 2020, and frontline healthcare workers were prioritized to receive the vaccine. To date, more than 80% of HCWs in Qatar have been received both doses of the Pfizer-BNT162b2 or Moderna-mRNA1273 vaccine. These vaccines are highly effective in preventing confirmed infection and nearly 100% effective in preventing critical disease or death [3–5]. However, breakthrough infections do occur in fully vaccinated persons [6].

We undertook this study to determine the factors associated with SARS-CoV-2 breakthrough infection after completion of a full course of vaccination. This study was not intended or designed to determine the vaccine effectiveness in HCWs.

## Methods

### Study population

We identified all healthcare workers at Hamad Medical Corporation in Qatar who had received 2 doses of Pfizer-BNT162b2 or Moderna-mRNA1273 vaccine between December 20, 2020 and May 18 2021, and had at least one SARS-CoV-2 RT-PCR positive on a nasopharyngeal swab specimen >14 days after the second dose. For each case thus identified, we identified one control with a negative test after December 20, 2020. Controls were matched on age, sex, nationality, job family and date of SARS-CoV-2 testing. We excluded those with a prior positive test and temporary workers.

### Covariates definitions

We performed chart reviews of each case and control to determine the presence of comorbidities. Presence of at least one physician note confirming the diagnosis was used to define the presence of a comorbidity. We conducted telephone interviews of cases and controls to ascertain presence of symptoms, any exposure to a confirmed case, and the accommodation type of the HCWs. Job family with HMC was ascertained through Human Resources data and categorized into Nursing and Midwifery, Physicians, Allied Health Professionals, Clinical Support Services, Administration, and Non-clinical Support Services. Accommodation status was categorized into single person housing, shared accommodation with immediate family members, and shared accommodation with non-family members.

### Statistical analyses

Baseline characteristics of vaccinated HCW with or without SARS-CoV-2 infection were compared using Chi-Square test, McNemar test or Mann–Whitney U test. Cox proportional hazards model was used to calculate the hazards ratios and 95% confidence intervals to determine the factors associated with breakthrough infection.

### Ethics statement

The study was approved by the Institutional Review Board at Hamad Medical Corporation (Protocol MRC-05-182). A waiver of informed consent was granted since data were collected as part of a national public health emergency response.

## Results

Among 22,247 HCW who had received both doses of the vaccine, we identified 164 HCW who met the criteria for breakthrough infection and matched them to 164 controls without breakthrough infection. Median age (IQR) was 39 (33,47) years, 50.6% were male, and 3.7% were Qatari nationals. **(Table 1)** HCWs in the nursing and midwifery job family constituted the largest group with breakthrough infection (41.5%), followed by allied health professionals (20.7%), and physicians (15.9%). Among 92 HCW in the breakthrough infection group with a history of contact with a confirmed infection, the spouse was identified as the most common positive contact (26.8%), followed by a patient (14%). **(Table 1)**

**Table 1:** Baseline characteristics of persons with documented breakthrough SARS-CoV-2 infection after vaccination compared with those who were vaccinated and not infected.

	Vaccinated with breakthrough infection, N (%)	Vaccinated Uninfected, N (%)	P-value
	N = 164	N = 164	
Median age (IQR) years	39 (33,47)	39 (33,47)	1
Male sex	83 (50.6)	83 (50.6)	1
Nationality			
Qatari	6 (3.7)	6 (3.7)	1
Non-Qatari	158 (96.3)	158 (96.3)	1
Body mass index, kg/m^2^, mean (SD)	28.37 ± 13.33	27.79 ± 5.1	0.56
< = 25	41 (30.6)	49 (32)	0.32
25–30	59 (44)	58 (37.9)	0.91
>30	34 (25.4)	46 (30.1)	0.12
Missing	30 (18.3)	11 (6.7)	
Comorbidity count			
None	105 (64)	98 (59.8)	0.43
1–2	54 (32.9)	59 (36)	0.56
≥3	5 (3.05)	7 (4.3)	0.56
Current smoker	20 (12.2)	17 (10.4)	0.60
Comorbidities			
Hypertension	24 (14.6)	17 (10.4)	0.2
Diabetes	17 (10.4)	18 (11)	1
Cardiovascular disease (MI, stroke, CHF)	8 (4.9)	5 (3)	0.55
Hyperthyroidism	8 (4.9)	10 (6.1)	0.80
Chronic lung disease (Asthma, COPD)	7 (4.3)	5 (3)	0.77
Hyperlipidemia	6 (3.7)	11 (6.7)	0.33
Chronic liver disease	3 (1.8)	2 (1.2)	1
Cancer diagnosis	2 (1.2)	4 (2.4)	0.69
Chronic kidney disease	1 (0.6)	1 (0.6)	1
Symptomatic at baseline	131(79.9)	28 (17.1)	<0.0001
Job family			
Nursing and Midwifery	68 (41.5)	68 (41.5)	1
Allied Health Professionals	34 (20.7)	34 (20.7)	1
Physicians	26 (15.9)	26 (15.9)	1
Non-clinical Support Services	15 (9.1)	15(9.1)	1
Administration	14 (8.5)	14 (8.5)	1
Clinical Support Services	7 (4.3)	7 (4.3)	1
Contact with confirmed case			
Spouse	44 (26.8)	9 (5.5)	<0.0001
Patients	23 (14)	5 (3)	<0.0001
Other family member	17 (10.4)	6 (3.7)	0.017
Colleagues	8 (4.9)	13 (7.9)	0.26
No history of contact with confirmed case	72 (43.9)	131 (79.9)	<0.0001
Accommodation status			
Family housing	116 (72)	96 (84.2)	0.02
Shared with non-family members	23 (14.3)	4 (3.5)	<0.0001
Single housing	22 (13.7)	14 (12.3)	0.16

In multivariate regression analyses, history of contact with a confirmed case (HR 1.58; 95% CI 1.13–2.20) and presence of symptoms (HR 3.24; 95% CI 2.07–5.05 for 1–2 symptoms; HR 3.66; 95% CI 2.34–5.73 for ≥3 symptoms) were associated with a higher risk of being diagnosed with breakthrough infection. Compared with Nursing and Midwifery staff, all other job families except Allied Health Professionals were at a higher risk of breakthrough infection. **(Table 2)**

**Table 2:** Factors associated with breakthrough infection (Cox regression model).

	Hazards ratio (95% confidence interval)	P-value
Age (per 10 years increase)	1.02 (0.61–1.68)	0.96
Gender (comparator: female sex)	1.26 (0.51–3.16)	0.62
Nationality (comparator: non-Qatari)	2.49 (0.44–14.00)	0.30
Job family (comparator: Nursing and Midwifery)		
Allied Health Professionals	2.99 (0.94–9.50)	0.06
Clinical Support Services	9.15 (1.32–63.64)	0.03
Administration	4.10 (1.13–14.90)	0.03
Physicians	6.27 (1.20–32.82)	0.03
Non-clinical Support Services	4.95 (1.00–23.45)	0.04
Body mass index <30 kg/m^2^ (comparator: ≥30)	2.99 (1.20–7.48)	0.02
Current smoker (comparator: never/ former smoker)	2.47 (0.69–8.90)	0.17
Number of comorbidities (comparator: None)		
1–2	1.48 (0.59–3.75)	0.41
3 or more	0.28 (0.02–4.11)	0.35
Contact with confirmed case (comparator: not exposed)	9.25 (3.67–23.28)	<0.0001
Accommodation status (comparator: Single/Family accommodation)	4.34 (0.74–25.46)	0.10
Symptomatic at baseline (comparator: None)		
1–2	30.28 (10.19–90.00)	<0.0001
≥ 3	112.58 (28.82–439.82)	<0.0001

A comparison of baseline characteristics of the 164 cases and the entire group of vaccinated uninfected HCWs (N = 22,247) is provided in **Table 3.**

**Table 3:** Baseline characteristics of persons with documented SARS-CoV-2 infection after vaccination compared with those who were vaccinated and not infected.

	Vaccinated Infected	Vaccinated Uninfected	P-value
	164	22,247	
Median age (IQR) years	39 (33,46)	38 (33,46)	0.88
Male sex	83	11221	0.97
Nationality			0.007
Qatari	6	2221	
Non-Qatari	158	20026	
Job family			<0.0001
Administration	14	2977	
Allied health professional	34	4186	
Clinical support	7	1038	
Executive leadership	0	22	
Nursing and midwifery	68	8682	
Physicians	26	2967	
Support services	15	2361	
Vaccine type			0.002
Pfizer-BNT162b2	164	21052	
Moderna-mRNA-1273	0	1195	

## Discussion

Our data from the largest integrated healthcare network in Qatar confirm our previous findings of a very low rate of breakthrough infections from one of the largest integrated healthcare networks in the United States. However, the factors associated with breakthrough infections are different in the two populations. In the United States Veterans population, we found increasing age to be associated with a higher risk of breakthrough infection. We did not have information on presence of symptoms or history of exposure in that study. In the current study, which was limited to HCWs, age was not independently associated with a higher risk of breakthrough infection. Of note, the median of the HCWs in the current study was 39 years compared with 73 years in the United States Veterans study.

A history of contact with a confirmed case and presence of symptoms were independently associated with higher risk. It is quite possible that these groups were more likely to be tested for SARS-CoV-2 infection because of these factors, in which case the number of cases of breakthrough infections identified would represent only a fraction of the actual cases. This limitation is largely mitigated in our study due to the fact that the HCWs were often tested as part of routine screening. An intriguing finding is the higher risk among almost all job families compared with nurses. This could represent a true lower risk among nurses due to better adherence with infection control measures and the fact that a large proportion of nurses lived in single or family accommodations. Conversely, clinical and non-clinical support staff were more likely to live is shared accommodation with non-family members, thus increasing their risk of infection.

Interestingly, presence of comorbidities was not associated with a higher risk of breakthrough infection. Multiple comorbidities have been reported to be associated with a higher risk of SARS-CoV-2 infection and poorer clinical outcomes in infected persons. The reason for a lack of association with comorbidities is unclear for persons developing breakthrough infections.

We noted a larger proportion of infections among persons vaccinated with the Pfizer-BNT-162b2 vaccine and none among the recipients of the Moderna-mRNA-1273 vaccine. Vaccination campaign in Qatar started with the Pfizer-BNT-162b2 vaccine with the Moderna-mRNA-1273 being introduced later. The difference in cases with different vaccines is largely due to the predominance of Pfizer-BNT vaccine and much shorter follow up period for Moderna vaccine, which was introduced later.

Strengths of our study include evaluation of a large integrated healthcare system with frequent and systematic testing of the HCWs, single accredited laboratory for testing of all samples, availability of all vaccination and testing data through the national pandemic response framework, and review of medical records and subject interviews to confirm clinical and social variables. Limitations include relatively small number of outcomes of interest, relatively unique demographic characteristics of the population which may not be generalizable to other countries, and relatively short follow up.

In conclusion, presence of symptoms and contact with a confirmed case are major risk factors for breakthrough SARS-CoV-2 infection after vaccination, and these groups should be prioritized for screening even after full vaccination.

## References

[1] Butt AA, Khan T, Yan P, Shaikh OS, Omer SB, Mayr F. Rate and risk factors for breakthrough SARS-CoV-2 infection after vaccination. J Infect. 2021;83(2):237–79. Epub 2021/05/31. doi: 10.1016/j.jinf.2021.05.021 ; PubMed Central PMCID: PMC8159711. 34052241 PMC8159711

[2] Alajmi J, Jeremijenko AM, Abraham JC, Alishaq M, Concepcion EG, Butt AA, et al. COVID-19 infection among healthcare workers in a national healthcare system: The Qatar experience. International journal of infectious diseases: IJID: official publication of the International Society for Infectious Diseases. 2020;100:386–9. Epub 2020/09/20. doi: 10.1016/j.ijid.2020.09.027 ; PubMed Central PMCID: PMC7493727. 32949777 PMC7493727

[3] Dagan N, Barda N, Kepten E, Miron O, Perchik S, Katz MA, et al. BNT162b2 mRNA Covid-19 Vaccine in a Nationwide Mass Vaccination Setting. N Engl J Med. 2021;384(15):1412–23. Epub 2021/02/25. doi: 10.1056/NEJMoa2101765 ; PubMed Central PMCID: PMC7944975. 33626250 PMC7944975

[4] Abu-Raddad LJ, Chemaitelly H, Butt AA, National Study Group for C-V. Effectiveness of the BNT162b2 Covid-19 Vaccine against the B.1.1.7 and B.1.351 Variants. N Engl J Med. 2021;385(2):187–9. Epub 2021/05/06. doi: 10.1056/NEJMc2104974 ; PubMed Central PMCID: PMC8117967. 33951357 PMC8117967

[5] Butt AA, Omer SB, Yan P, Shaikh OS, Mayr FB. SARS-CoV-2 Vaccine Effectiveness in a High-Risk National Population in a Real-World Setting. Ann Intern Med. 2021: doi: 10.7326/m21-1577 Epub 2021/07/20. ; PubMed Central PMCID: PMC8381771. 34280332 PMC8381771

[6] Hacisuleyman E, Hale C, Saito Y, Blachere NE, Bergh M, Conlon EG, et al. Vaccine Breakthrough Infections with SARS-CoV-2 Variants. N Engl J Med. 2021;384(23):2212–8. Epub 2021/04/22. doi: 10.1056/NEJMoa2105000 ; PubMed Central PMCID: PMC8117968. 33882219 PMC8117968

